# Secure image encryption algorithm using chaos-based block permutation and weighted bit planes chain diffusion

**DOI:** 10.1016/j.isci.2023.108610

**Published:** 2023-12-02

**Authors:** Heping Wen, Yiting Lin, Shenghao Kang, Xiangyu Zhang, Kun Zou

**Affiliations:** 1 University of Electronic Science and Technology of China, Zhongshan Institute, Zhongshan 528402, China; 2 School of Information and Communication Engineering, University of Electronic Science and Technology of China, Chengdu 611731, China; 3 School of Computer Science and Engineering (School of Cyber Security), University of Electronic Science and Technology of China, Chengdu 611731, China; 4 School of Automation, Guangdong University of Technology, Guangzhou 510006, China

**Keywords:** Physics, Quantum theory

## Abstract

Aiming at the problem of insufficient security of image encryption technology, a secure image encryption algorithm using chaos-based block permutation and weighted bit planes chain diffusion is proposed, which is based on a variant structure of classical permutation-diffusion. During the permutation phase, the encryption operations of dividing an image into sub-block, block scrambling, block rotation and block inversion, negative-positive transformation, color component shuffling are performed sequentially with chaotic sequences of plaintext association. In the chain diffusion stage, different encryption strategies are adopted for the high and low 4-bit planes according to the weight of image information. Theoretical analyses and empirical results substantiate that the algorithm conforms to the cryptographic requirements of confusion, diffusion, and avalanche effects, while possessing excellent numerical statistical properties with a large cryptographic space. Therefore, the cryptanalysis-propelled security enhancement mechanism proposed in this paper effectively amplifies the aptitude of the algorithm to withstand cryptographic attacks.

## Highlights

• The proposed encryption algorithm can effectively resist the chosen-plaintext attack
• Bit level is used as the basic unit of image encryption to ensure higher level of security
• Chaos-based dynamic block permutation achieves good confusion with low complexity
• The receiver can decrypt the message without transmitting additional plaintext related keys

## Graphical abstract

**Figure:**
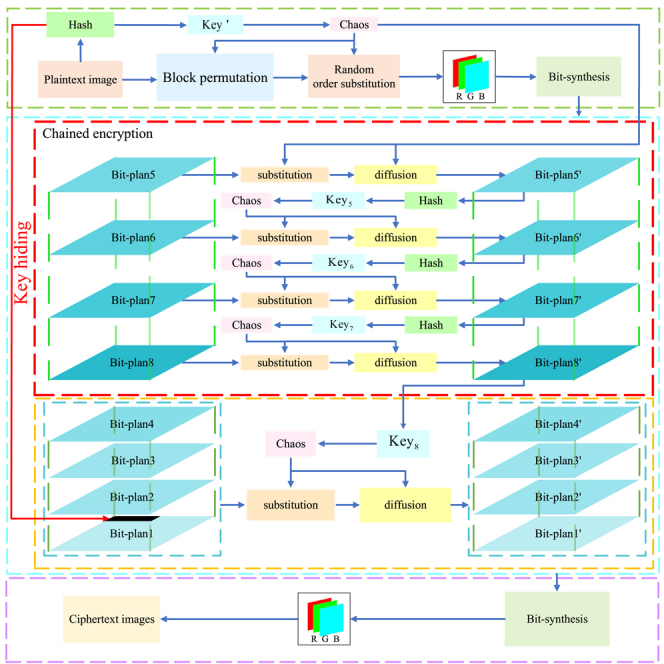

## Introduction

Today, a variety of emerging information technologies are developing by leaps and bounds. As individuals revel in the dividends of information technologies, latent security concerns are progressively unveiled. The exploration of reliable and efficient security algorithms is gaining significance in the big data environment.^1^^,^^2^^,^^3^ In the era of big data, the secure transmission of digital images has emerged as a pivotal concern, garnering substantial academic attention. Ensuring that these images remain confidential during transmission, safeguarding against theft or leakage has become a prominent research focus.^4^^,^^5^^,^^6^ However, due to the distinctiveness of digital images,^7^^,^^8^^,^^9^ such as high correlation of adjacent pixel points, scattering distribution of critical information, and high information redundancy, cryptographic protection using traditional text encryption algorithms struggles to meet real-time performance requirements. Furthermore, chaos itself exhibits inherent qualities^10^^,^^11^^,^^12^^,^^13^ such as high sensitivity to initial conditions and control parameters, excellent pseudo-randomness, ergodicity, and long-term unpredictability of orbits, and it has many similarities with confusion, diffusion, and so on in cryptography.^14^^,^^15^^,^^16^^,^^17^ Hence, in the context of the big data era, it is crucial to explore the image encryption algorithm that is firmly rooted in chaos theory.^18^^,^^19^^,^^20^^,^^21^

Throughout the international research status,^22^^,^^23^^,^^24^^,^^25^ exploration of chaotic image encryption algorithms^26^^,^^27^^,^^28^^,^^29^ has been going through more than 20 years. In as early as 1998, Fridrich^30^ firstly reported the use of chaotic system to encrypt digital images. In recent years, numerous scholars have dedicated their efforts to this field, leading to significant progress and enrichment in the theory and methods of chaotic image encryption.^31^^,^^32^^,^^33^ Various new mechanisms and methods are introduced into chaotic image encryption to enhance the security of the algorithm and elevate the performance of secure transmission. Zheng et al.^34^ in 2022 outlined an image encryption technique that uses cascaded chaotic maps and an extended zigzag transform. The simulation results demonstrate that the algorithm provides fast encryption, high security, and effective protection to withstand a broad spectrum of attacks. In 2023, Jiang et al.^35^ introduced an image encryption algorithm based on a two-dimensional Chebyshev logistic infinite collapse map. The results provide a solid foundation and practical solutions for chaos generation and image encryption.^36^^,^^37^^,^^38^^,^^39^ These achievements mean that many encryption algorithms have shown satisfactory results in several areas,^40^^,^^41^^,^^42^^,^^43^ which have significantly advanced the field of information security technology.^44^^,^^45^^,^^46^^,^^47^ However, as society develops, the exponential growth of information poses significant challenges to previous algorithms.^48^^,^^49^^,^^50^^,^^51^ The limitations of these algorithms become increasingly prominent.^52^^,^^53^^,^^54^^,^^55^

Most previous algorithms perform operations at the pixel level, and the granularity of their encryption units is coarse. To meet this challenge, there has been significant interest in chaotic image encryption methods employing bit plane decomposition^56^^,^^57^^,^^58^ in recent years. Numerous encryption algorithms based on bit planes have been proposed, garnering widespread attention. Bit planes decomposition is an algorithm that divides a digital image by bit to obtain eight subgraphs and encrypts each of these subgraphs independently. Although some progress has been made in chaotic image encryption research, the designed algorithms still have certain limitations due to the existing defects in bit encryption. In 2019, Shafique et al.^59^ introduced an encryption algorithm that incorporates binary bit plane extraction and multiple chaotic maps. The system comprises two parts, bit-level permutation of the high 4-bit planes and bitwise XOR diffusion. They presented several security analyses and experimental simulations demonstrating the system’s ability to withstand various attacks. However, the study found that the diffusion and permutation components could be separately attacked using a divide-and-conquer approach, highlighting their inherent vulnerabilities.^60^ In 2020, a novel image encryption algorithm leveraging DNA coding and spatiotemporal chaos was unveiled.^61^ The algorithm involves a sequential implementation of pixel diffusion, DNA encoding, DNA base permutation, and DNA decoding to create a cipher image from a plaintext image. However, this algorithm ultimately represents a fusion of fixed DNA base permutation and bit complement. Consequently, the algorithm can be compromised by chosen-plaintext attacks and chosen-ciphertext attacks. From a security perspective, existing bit-level chaotic encryption algorithms need further improvement, mainly because 1) the present algorithms are susceptible to chosen-plaintext attacks or chosen-ciphertext attacks as the key employed for generating chaotic sequences lacks correlation with the original image; 2) the granularity of encryption units of existing algorithms is coarse; 3) existing bit planes decomposition algorithms do not consider the correlation between each sliced plane after decomposition.

This paper presents a secure image encryption algorithm that utilizes chaos-based block permutation and weighted bit planes chain diffusion. The proposed algorithm is based on a variant structure of classical permutation-diffusion, which first performs a block permutation based on chaotic sequences yet executes a bit-level chain diffusion. During the permutation phase, the encryption operations of dividing an image into sub-block, block scrambling, block rotation and block inversion, negative-positive transformation, color component shuffling are performed sequentially based on the chaotic sequences of plaintext association. In the bit-level diffusion stage, the high and low 4-bit planes are encrypted according to the information weight contained in the image. A chain-substitution-diffusion encryption with bit-by-bit planes is adopted for the high 4-bit planes, followed by a lightweight encryption for the low 4-bit planes. Among them, the intermediate ciphertext association mechanism is used in all the diffusion encryption processes. Thus, the algorithm possesses the prowess to fend off both known-plaintext attack and chosen-plaintext attack due to the closed-loop feedback of plaintext and ciphertext. The primary contributions and innovations of this study are delineated as follows.

a. The existing image encryption algorithms are not structured rationally enough, which leads to their insufficient security against plaintext-type attacks. For this reason, this color image encryption algorithm proposes a plaintext and intermediate ciphertext association mechanism and also adopts chain diffusion to effectively enhance the resistance to cryptographic attacks.
b. Pixel-level image encryption is so coarse in granularity that it is not secure enough, and traditional bit-level encryption is too complex to meet the efficiency requirements. To cope with these challenges, this paper proposes a new strategy. We adopt an elastic processing unit in the weighted bit plane, which effectively balances the tension between security and efficiency.
c. Different from the traditional permutation methods, this paper designs a new block permutation based on several algebraic operations. Since each module adopted in the permutation is all low-complexity units that are easily implemented by computers, they have good confusion characteristics. The experimental results also effectively support the feasibility of the block permutation method in this paper.
d. In image encryption using plaintext correlation, extra channels must be communicated to transmit the feature values of the plaintext. In contrast, we embed the feature values associated with the plaintext and the intermediate ciphertext into the cipher image, and the receiver can achieve normal decryption without additional key exchange, which ensures the usability in practical applications.

The remaining sections of this paper are organized as follows. Section Related theory briefly outlines the relevant theory behind the proposed algorithm. Section Proposed encryption algorithm explains the precise details of our encryption algorithm. Section Experimental results and analysis discussion presents experimental results and analysis discussion. The final section concludes the study.

### Related theory

#### The used chaotic system

##### 2D logistic-sine-coupling map

The chaos^62^ utilized in this research is sourced from two established one-dimensional chaotic maps, namely the logistic map and the sine map. By coupling the logistic map and the sine map, we can acquire a new chaotic map of considerable complexity, namely the 2D-LSCM, which can be determined as follows:

(Equation 1) $$\left\{ \begin{array}{l} a_{i+1}=\sin\left(\pi \left(4\theta a_{i}\left(1-a_{i}\right)+\left(1-\theta \right)\sin\left(\pi b_{i}\right)\right)\right)\\ b_{i+1}=\sin\left(\pi \left(4\theta b_{i}\left(1-b_{i}\right)+\left(1-\theta \right)\sin\left(\pi a_{i+1}\right)\right)\right) \end{array}\right.$$

where θ ∈ [0,1] is the control parameter. As the definition suggests, the 2D-LSCM is generated by coupling the logistic and sine maps; the results are further subjected to sine transformation and expanding from one dimension to two. Through this process, the intricacies of the logistic map and the sine map are intertwined, resulting in a complex chaotic behavior.

##### NIST test results of chaos

A collection of 16 unique test sets is provided by the NIST-800-22 test suite, with the aim of assessing binary sequences generated by cryptographic or pseudo-random number generators that depend on random values and lengths. Notably, all sequences intended for encryption have passed this test correctly, and some of the results are presented in the Table 1.

**Table 1:** The test results for NIST-800-22

Statistical	p values		
	Sequence1	Sequence2	Results
Frequency (Monobit) Test	0.534146	0.739918	successful
Block-frenquency test	0.739918	0.350485	successful
Cumulative-sums test	0.000954	0.122325	successful
Runs test	0.911413	0.739918	successful
Longest-run test	0.350485	0.911413	successful
Binary matrix rank test	0.534146	0.534146	successful
Discrete Fourier transform test	0.534146	0.534146	successful
Non-overlapping templates test	0.000439	0.008879	successful
Overlapping templates test	0.017912	0.122325	successful
Maurer’s universal statistical test	0.035174	0.534146	successful
Approximate entropy test	0.534146	0.991468	successful
Random-excursions test(x = −4)	0.022503	0.048716	successful
Random-excursions variant test(x = −9)	0.022503	0.122325	successful
Serial test-1	0.911413	0.739918	successful
Serial test-2	0.066882	0.213309	successful
Linear-complexity test	0.739918	0.911413	successful

##### 0-1 Test results of chaos

**Figure 1:**
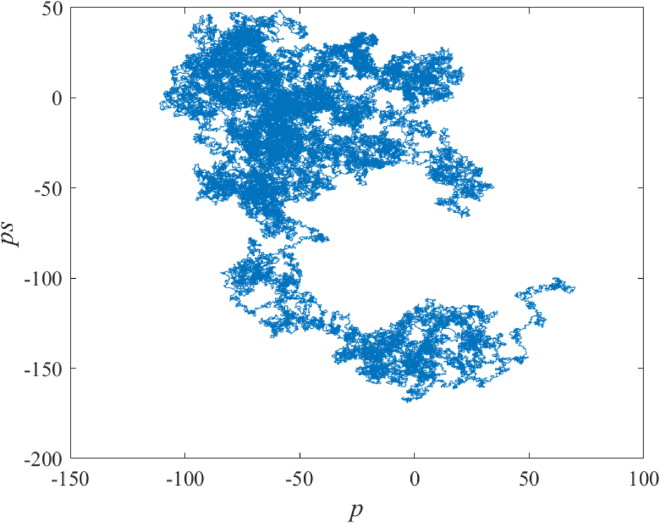
0–1 Gottwald Melbourne test

The 0–1 Gottwald Melbourne test is a tool that calculates parameters very close to 0 or 1 to accurately distinguish between regular and chaotic motion. Our team used the 0–1 Gottwald Melbourne test to obtain 10,000 results, which reflect the average value of 0.9979, exhibiting the remarkable performance of the chaotic system. The test results are illustrated in the following Figure 1.

#### Bit planes decomposition

**Figure 2:**
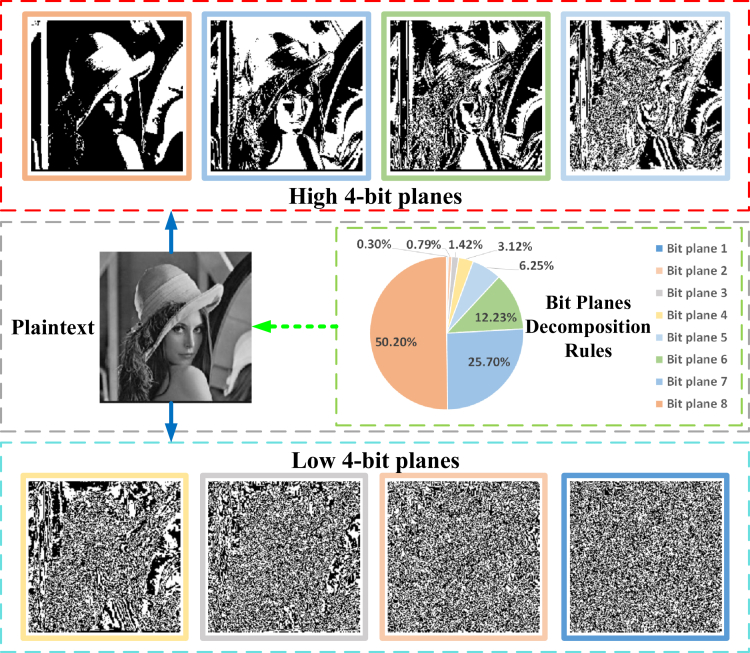
Information ratio of pixels occupied by 8-bit planes

A digital image is created by converting an analog image into a digital format, where the pixels serve as the basic element that can be stored and processed by a digital computer or circuit. In computing, a bit is a unit of information and the smallest unit of measurement for bits and information within a binary number. The range of pixel values in an image is $\left[2^{0}-1\text{,}2^{8}-1\right]$. Bit plane decomposition involves converting the pixel values of a digital image into binary form and then dividing the binary representation into eight-bit planes. Taking a digital image *P* as an example, the bit planes decomposition can be expressed as

(Equation 2) $$P=\sum_{k=1}^{8}2^{k-1}P_{k}=P_{1}+2P_{2}+2^{2}P_{3}+2^{3}P_{4}+2^{4}P_{5}+2^{5}P_{6}+2^{6}P_{7}+2^{7}P_{8}$$

where $P_{k}$ denotes the k−th bit plane, k=[1,2,3,…,8], $P\left(i,j\right)\in Z_{256}$, $P_{k}\left(i,j\right)\in Z_{2}$; $P_{8}$ denotes the highest bit planes, and $P_{1}$ denotes the lowests bit planes. Taking the grayscale image of “Lena” as an example, the bit planes decomposition diagram is shown in Figure 2.

#### Block permutation

**Figure 3:**
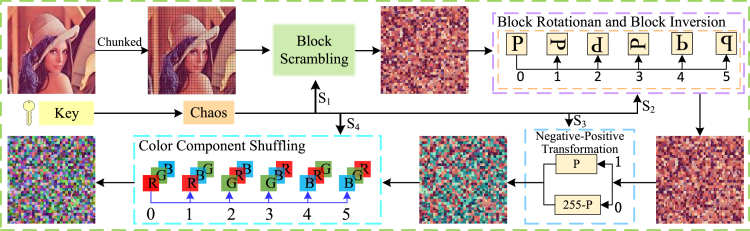
Block permutation flowchart

Standard scrambling algorithms, which simply rearrange the pixel positions within the primitive image, can be easily deciphered and are susceptible to chosen-plaintext attacks. To address this problem, this paper introduces a new scrambling algorithm that uses chaotic sequences based on the plaintext feedback mechanism, as shown in Figure 3. Firstly, the image feature values are extracted as chaotic keys to generate an initial pseudo-random sequence. Then, the original image and initial chaotic sequence are preprocessed. Finally, different encryption rules can be selected according to the preprocessed sequences, and the generated cipher images can be obtained by block scrambling, block rotation and block inversion, negative-positive transformation, and color component shuffling. The empirical data demonstrate that the permutation mechanism proposed in this manuscript significantly enhances the algorithm’s resilience against attacks.

### Proposed encryption algorithm

**Figure 4:**
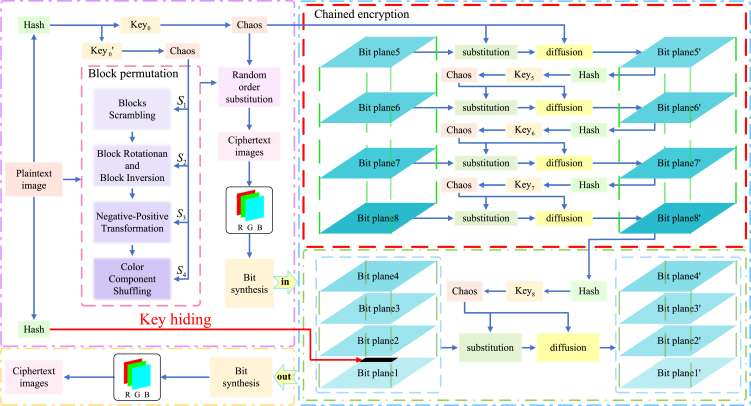
Diagram depicting the procedural flow of the proposed encryption algorithm

The proposed encryption algorithm is specified based on the following four sections: the first part, key generation and initial value scrambling; the second part, an explanation of the user-defined “chain function”; the third part, the process of image encryption; and the fourth part, the image decryption process. In this paper, assuming an original image *P* is input, the process of encrypting into a cipher image *C* consists of three modules. First, the original image is block permuted. Next, a random order substitution is executed on the permuted image. Finally, the resulting intermediate cipher image is layered according to Equation 2, after which the final cipher image can be obtained. The overall block diagram illustrating the flow of the encryption algorithm is depicted Figure 4.

#### Key generation and initial value scrambling

This algorithm utilizes the MD5 hash function to establish the correspondence between plaintext and ciphertext. The hash function’s one-way property and anti-collision feature substantially enhance the algorithm’s capability to withstand attacks from chosen-plaintext attacks and known-plaintext attacks.

Step1: read image feature values

After reading the image features by MD5 function, we can get a 32-bit hexadecimal number with fixed bit length, and each bit is represented as h(x)∈{0,1,2,…,14,15}, where x=[1,2,3,…,31,32].

Step2: disturb the initial value

After obtaining the eigenvalues, the initial value of the chaotic map is perturbed. This allows different images to correspond to different key sequences and improves their resistance to differential attacks. The specific procedure is defined as

(Equation 3) $$\left\{ \begin{array}{ll} key_{1}=h\left(1\right)⊕h\left(5\right)⊕h\left(9\right)⊕h\left(13\right)⊕h\left(17\right)⊕h\left(21\right)⊕h\left(25\right)⊕h\left(29\right)+0.1 & \left(a\right)\\ key_{2}=h\left(2\right)⊕h\left(6\right)⊕h\left(10\right)⊕h\left(14\right)⊕h\left(18\right)⊕h\left(22\right)⊕h\left(26\right)⊕h\left(30\right)+0.1 & \left(b\right)\\ key_{3}=h\left(3\right)⊕h\left(7\right)⊕h\left(11\right)⊕h\left(15\right)⊕h\left(31\right)⊕h\left(23\right)⊕h\left(27\right)⊕h\left(31\right)+0.1 & \left(c\right)\\ key_{4}=h\left(4\right)⊕h\left(8\right)⊕h\left(12\right)⊕h\left(16\right)⊕h\left(20\right)⊕h\left(24\right)⊕h\left(28\right)⊕h\left(32\right)+0.1 & \left(d\right) \end{array}\right.$$

where ⊕ denotes the XOR operator; $key_{1},key_{2},key_{3},key_{4}$ denote the scrambled keys.

#### Chain function

##### Chain encryption function

For ease of description, we define the details of the operation for each plane as a self-named function C=Chain_encrypt(P,key), where *P* represents the plain image, key denotes the initial value utilized to generate the chaotic sequence for encrypting the image plane, and *C* denotes the cipher image. The function contains two parts: sequence preprocessing and permutation-diffusion encryption. Take the example of encrypting an image with size H×W as described below:

Step1: preprocess chaotic sequence

For the chain encryption module, a total of two chaotic sequences are required. Key is introduced into the 2D-LSCM chaotic system, and the initial chaotic sequences $R_{1}$ and $R_{2}$ are obtained after iterating H×W times. The corresponding sorted indexes indexH and indexW can be described as

(Equation 4) $$\left\{ \begin{array}{c} \left[S_{1},indexH\right]=sort\left(R_{1}\right)\\ \left[S_{2},indexW\right]=sort\left(R_{2}\right) \end{array}\right.$$

where sort(·) function represents the sorting of each sequence value in the input sequence from lowest to highest, $S_{1}$ and $S_{2}$ indicate the result of reordering the sequences, and indexH and indexW indicate sorted indexes.

Step2: permutation and diffusion encryption

By sequence indexH and indexW, performing row-column permutation on the layered images to be encrypted results in permuted image $C_{1}$. Then it is bit-level diffused to secure the cipher image *C*. The operation is shown as

(Equation 5) $$\left\{ \begin{array}{l} C_{1}\left(m,n\right)=P\left(indexH\left(m\right),indexW\left(n\right)\right)\\ C\left(m,n\right)=C_{1}\left(m,n\right)⊕R\left(m,n\right) \end{array}\right.$$

where m=[1,2,…,H] and n=[1,2,…,W].

##### Chain decryption function

For the convenience of a detailed exposition on the decryption process in subsequent sections, the definition of the chain decryption function P=Chain_decrypt(C,key) will be given, where *P* denotes the recovered image, *C* denotes the image that is waiting to be decrypted, and key denotes the initial key to generate the desired chaotic sequences. After putting the key into the chaotic system, it iterates H×W times to obtain two chaotic sequences $R_{1}^{\prime }$ and $R_{2}^{\prime }$. Substituted into the Equation 4, the sorted index sequence $indexH^{\prime }\left(m\right)$ and $indexW^{\prime }\left(n\right)$ can be obtained, respectively. The decryption process is shown in Equation 6.

(Equation 6) $$\left\{ \begin{array}{l} C_{1}\left(m,n\right)=C\left(m,n\right)⊕R^{\prime }\left(m,n\right)\\ P\left(indexH^{\prime }\left(m\right),indexW^{\prime }\left(n\right)\right)=C_{1}\left(m,n\right) \end{array}\right.$$

#### Encryption process

In this section, an original image *P* of size H×W will be used as an example to illustrate the encryption process. The first encryption is a block permutation operation after chunking the original image. In this article, we choose to divide the image into small blocks with a length of 8, and the process is as follows.

Step1: preprocess image and sequence

Initially, the plaintext image *P* is divided into segments and transformed into a matrix format, ensuring that the matrix dimensions are multiples of 8 for both rows and columns. If there are not enough elements in the matrix, the zeroes are filled. Then, the hash eigenvalues of the image *P* are read and $key_{1},key_{2},key_{3},key_{4}$ are obtained as chaotic initial values according to Equation 3. The four initial values are iterated by the 2D-LSCM system to obtain four pseudo-random sequences $S_{1},S_{2},S_{3},S_{4}$. And they are preprocessed according to the Equation 7 to obtain sequences $S_{1}^{\prime },S_{2}^{\prime },S_{3}^{\prime },S_{4}^{\prime }$ that can be used for block scrambling operation. The pretreatment Equation 7 is described as

(Equation 7) $$\left\{ \begin{array}{l} S_{1}^{\prime }=\left\lfloor \left(S_{1}\times 10^{10}\right)\mathrm{mod}\left(\left(H\times W\right)/8^{2}\right)\right\rfloor \\ S_{2}^{\prime }=\left\lfloor \left(S_{2}\times 10^{10}\right)\mathrm{mod}\;6\right\rfloor \\ S_{3}^{\prime }=\left\lfloor \left(S_{3}\times 10^{10}\right)\mathrm{mod}\;2\right\rfloor \\ S_{4}^{\prime }=\left\lfloor \left(S_{4}\times 10^{10}\right)\mathrm{mod}\;6\right\rfloor \end{array}\right.$$

where ⌊·⌋ indicates rounding down, and mod(·) represents modulo operation.

Step2: block scrambling

**Figure 5:**
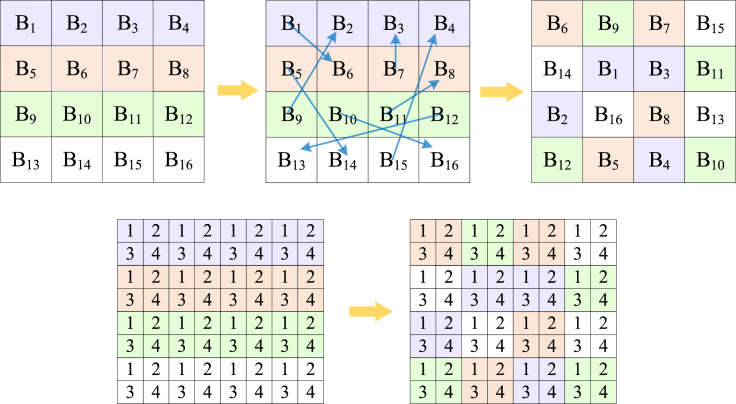
Diagram illustrating block scrambling

The specific diagram of block scrambling is shown in Figure 5. The operation of block scrambling is as follows:

(Equation 8) $$\left\{ \begin{array}{l} t=P\left(x,y,z,i\right)\\ P\left(x,y,z,i\right)=P\left(x,y,z,S_{1}^{\prime }\left(i\right)\right)\\ B\left(x,y,z,S_{1}^{\prime }\left(i\right)\right)=t \end{array}\right.$$

where x=[1,2,…,H], y=[1,2,…,W], $i=\left[1,2,\ldots ,H\times W/8^{2}\right]$, *z* signifies the dimension of the matrix, *t* indicates the intermediate variable, and B represents the matrix after block scrambling.

Step3: block rotation and block inversion

**Figure 6:**
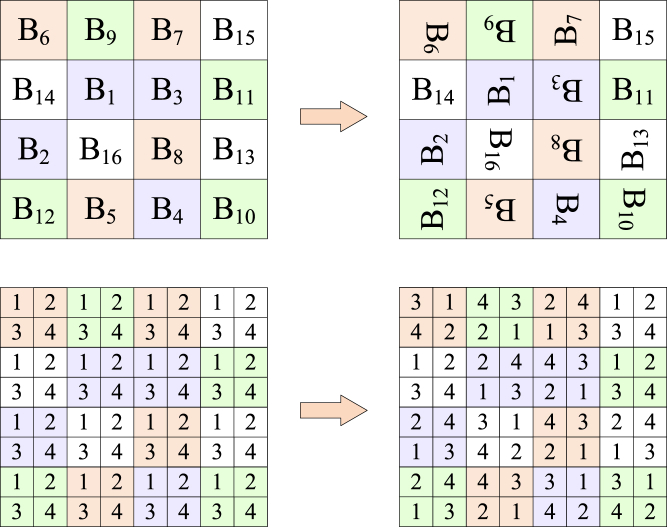
Schematic diagram of block rotation and block inversion

Based on the sequence $S_{2}^{\prime }$, block rotation and block inversion are performed on the data in the sub-block. Taking the matrix after block scrambling encryption as an example, the matrix after inverse encryption is obtained by using the method constructed in Algorithm 1, and the schematic of block rotation and inversion is shown in Figure 6.

Algorithm 1 **Block rotation and block inversion**

**Input:** pseudo-random sequence $S_{2}^{\prime }$, intermediate ciphertext CI, block side length Bsl, image length *H*, and image width *W*:

**Output:** block rotation and block inversion encrypt ciphertext CI

1: **for** x←1 to H/Bsl **do**

2: **for** y←1 to W/Bsl **do**

3: **for** a←1 to Bsl/2 **do**

4: **for** b←1 to Bsl/2 **do**

5: **if** $S_{2}^{\prime }=1$ **then**

6:  swap(CI(a,b,:,x,y),CI(Bsl+1−b,a,:,x,y));

7:  swap(CI(Bsl+1−b,a,:,x,y),CI(Bsl+1−a,Bsl+1−b,:,x,y));

8:  swap(CI(Bsl+1−a,Bsl+1−b,:,x,y),CI(b,Bsl+1−a,:,x,y));

9: **else if** $S_{2}^{\prime }=2$ **then**

10:  swap(CI(a,b,:,x,y,CI(Bsl+1−a,Bsl+1−b,:,x,y));

11:  swap(CI(a,b+Bsl/2,:,x,y),CI(Bsl+1−a,Bsl/2+1−b,:,x,y));

12: **else if** $S_{2}^{\prime }=3$ **then**

13:  swap(CI(a,b,:,x,y),CI(b,Bsl+1−a,:,x,y));

14:  swap(CI(b,Bsl+1−a,:,x,y),CI(Bsl+1−a,Bsl+1−b,:,x,y));

15:  swap(CI(Bsl+1−a,Bsl+1−b,:,x,y),CI(Bsl+1−b,a,:,x,y));

16: **else if** $S_{2}^{\prime }=4$ **then**

17:  swap(CI(a,b,:,x,y),CI(a,Bsl+1−b,:,x,y));

18:  swap(CI(Bsl/2+a,b,:,x,y),CI(Bsl/2+a,Bsl+1−b,:,x,y));

19: **else if** $S_{2}^{\prime }=5$ **then**

20:  swap(CI(a,b,:,x,y),CI(Bsl+1−a,b,:,x,y));

21:  swap(CI(a,Bsl/2+b,:,x,y),CI(Bsl+1−a,Bsl/2+b,:,x,y));

22: **end if**

23: **end for**

24: **end for**

25: **end for**

26: **end for**

Step4: negative-positive transformation

$S_{3}$ after the operation of taking modulo 2, the obtained $S_{3}^{\prime }$ has two values of 0 or 1, and their respective corresponding operations are shown as

(Equation 9) $$\left\{ \begin{array}{ll} B\left(x,y,z,i,j\right)=255-B\left(x,y,z,i,j\right) & if\;S_{3}^{\prime }=0\\ B\left(x,y,z,i,j\right)=B\left(x,y,z,i,j\right) & if\;S_{3}^{\prime }=1 \end{array}\right.$$

B represents the matrix after block rotation and block inversion.

Step5: color component shuffling

**Figure 7:**
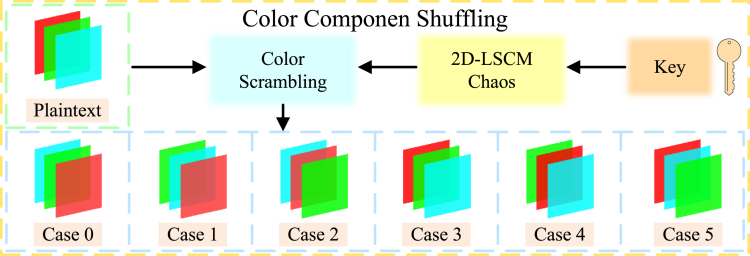
Schematic diagram of color component shuffling

Based on the sequence $S_{4}^{\prime }$, the corresponding RGB color transformation is applied to the processed matrix using Algorithm 2. The specific color component shuffling schematic is shown in Figure 7.

Algorithm 2 **Color component shuffling**

**Input:** pseudo-random sequence $S_{4}^{\prime }$, intermediate cipher image CI, block side length Bsl, image block length *H*, and image block width *W*

**Output:** block permutated image BC

1: **for** a←1 to H/Bsl **do**

2: **for** b←1 to W/Bsl **do**

3: **if** $S_{4}^{\prime }=1$ **then**

4: BC=swap(CI(:,:,2,a,b),CI(:,:,3,a,b));

5: **else if** $S_{4}^{\prime }=2$ **then**

6: BC=swap(CI(:,:,2,a,b),CI(:,:,3,a,b));

7: **else if** $S_{4}^{\prime }=3$ **then**

8: BC=swap(CI(:,:,2,a,b),CI(:,:,3,a,b));

9: **else if** $S_{4}^{\prime }=4$ **then**

10: BC=swap(CI(:,:,2,a,b),CI(:,:,3,a,b));

11: BC=swap(CI(:,:,2,a,b),CI(:,:,3,a,b));

12: **else if** $S_{4}^{\prime }=5$ **then**

13: BC=swap(CI(:,:,2,a,b),CI(:,:,3,a,b));

14: BC=swap(CI(:,:,2,a,b),CI(:,:,3,a,b));

15: **end if**

16: **end for**

17: **end for**

The matrix after block encryption is merged to get block permutated image BC(BlockCipher).

Next, it will be subjected to a random order substitution operation.

Step6: random order substitution

The $key_{1}$ and $key_{2}$ are substituted into the chaotic system iteration to obtain the sequences *S* and $S^{\prime }$. The index matrix *I* is achieved by arranging the sequence $S^{\prime }$. Random order substitution of cipher images is carried out using index matrix *I* with sequence *S*. The random order substitution can be explained as follows:

(Equation 10) $$Q_{I_{i,j},j}=\left\{ \begin{array}{ll} \left(BC_{I_{i,j},j}+BC_{I_{H,W},W}+\left\lfloor 2^{32}\times S_{I_{i,j},j}\right\rfloor \right)\mathrm{mod}\;256 & for\;i=1,j=1\\ \left(BC_{I_{i,j},j}+BC_{I_{i-1,W},W}+\left\lfloor 2^{32}\times S_{I_{i,j},j}\right\rfloor \right)\mathrm{mod}\;256 & for\;i=2∼W,j=1\\ \left(BC_{I_{i,j},j}+BC_{I_{i,j-1},j-1}+\left\lfloor 2^{32}\times S_{I_{i,j},j}\right\rfloor \right)\mathrm{mod}\;256 & for\;i=1∼W,j=2∼W \end{array}\right.$$

After obtaining image *Q*, it will be subjected to bit-layered and chained encryption.

Step7: bit planes decomposition

After reading the image *Q* and splitting it according to the three channels, three grayscale images $Q_{R}$, $Q_{G}$, and $Q_{B}$ are obtained, which are bit planes decomposition, respectively. It can be expressed as

(Equation 11) $$\left\{ \begin{array}{c} Q_{Rk}=bitget\left(Q_{R},k\right)\\ Q_{Gk}=bitget\left(Q_{G},k\right)\\ Q_{Bk}=bitget\left(Q_{B},k\right) \end{array}\right.$$

where function bitget(A,bit) denotes the bit value at position *k* in *A* is returned, $Q_{Rk}$, $Q_{Gk}$, and $Q_{Bk}$ denote the images obtained by layering $Q_{R}$, $Q_{G}$, and $Q_{B}$, and *k* denotes the k−th bit plane, k=[1,2,3,4,5,6,7,8].

Taking *R* channel as an example, the other two channels are the same. Specifically, the obtained 8 layered images are $Q_{R1},Q_{R2},Q_{R3},Q_{R4},Q_{R5},Q_{R6},Q_{R7},Q_{R8}$.

Step8: hide original image feature values in the first bit plane

In order to facilitate the decryption operation by the recipient, the hash value of the original image needs to be stored in the first line of layered image $Q_{R1}$. It is worth noting that, as shown by the analysis in Section Bit planes decomposition, the first layer of the bit plane contains very little information. Taking a 256 × 256 size image as an example, the proportion of the feature value in the original image is only 0.000586%. Even if the decrypted image is enlarged, it is difficult to observe the difference with the naked eye.

Step9: encrypt $Q_{R5}$ with $key_{1}$

(Equation 12) $$C_{R5}=Chain\_encrypt\left(Q_{R5},key_{1}\right)$$

Step10: chain encrypt $Q_{R6},Q_{R7},Q_{R8}$

After the fifth layer cipher image $C_{R5}$ is obtained, the hash eigenvalue is read and substituted into the Equation 3 (a), and the chaotic initial value $key_{5}$ used to encrypt the next layer is obtained. The encryption of the sixth layer bit plane is given below:

(Equation 13) $$C_{R6}=Chain\_encrypt\left(Q_{R6},key_{5}\right)$$

The encryption method for the images $Q_{R7},Q_{R8}$ is the same as earlier and can be expressed by the Equation 14.

(Equation 14) $$\left\{ \begin{array}{c} C_{R7}=Chain\_encrypt\left(Q_{R7},key_{6}\right)\\ C_{R8}=Chain\_encrypt\left(Q_{R8},key_{7}\right) \end{array}\right.$$

Step11: chain encrypt $Q_{R1},Q_{R2},Q_{R3},Q_{R4}$

For the low-order bit plane, only a small amount of image information is contained, so the same chaotic sequence will be used to encrypt the four layers of $Q_{R1},Q_{R2},Q_{R3},Q_{R4}$. Similarly, after reading the eigenvalues of $C_{R8}$ and performing the processing as in Equation 3 (a), the $key_{8}$ is obtained, and the encryption of these four layers can be expressed as

(Equation 15) $$\left\{ \begin{array}{c} C_{R1}=Chain\_encrypt\left(Q_{R1},key_{8}\right)\\ C_{R2}=Chain\_encrypt\left(Q_{R2},key_{8}\right)\\ C_{R3}=Chain\_encrypt\left(Q_{R3},key_{8}\right)\\ C_{R4}=Chain\_encrypt\left(Q_{R4},key_{8}\right) \end{array}\right.$$

Step12: compound bit planes

The encrypted images $C_{R1},C_{R2},C_{R3},C_{R4},C_{R5},C_{R6},C_{R7},C_{R8}$ are planes compounded as

(Equation 16) $$C_{R}=\sum_{i=1}^{8}2^{i-1}C_{Ri}=2^{0}C_{R1}+2^{1}C_{R2}+2^{2}C_{R3}+2^{3}C_{R4}+2^{4}C_{R5}+2^{5}C_{R6}+2^{6}C_{R7}+2^{7}C_{R8}$$

where $C_{R}$ denotes the final cipher image of the *R* channel after reduction.

Similarly, we can get the cipher image $C_{G},C_{B}$ after the chain encryption of *G* channel and *B* channel, and the resulting cipher image *C* is obtained after the reduction of three channels.

#### Decryption process

**Figure 8:**
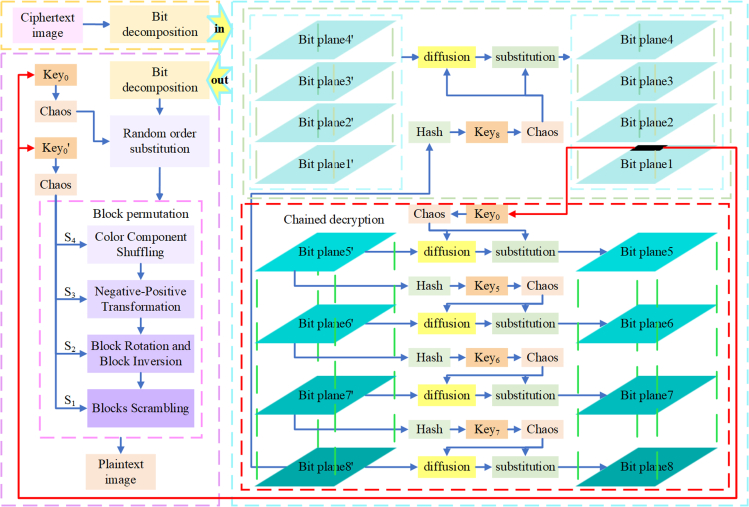
The flow chart of decryption

Decryption is the reverse process of encryption. The flow chart of decryption is shown in Figure 8. For simplicity, a brief description of the decryption process is as follows.

Step1: bit planes decomposition

After dividing the cipher image *C* into RGB three channels, we get $C_{R},C_{G},C_{B}$. Take the *R* channel as an example; the remaining two channels are the same. According to Equation 11, 8-bit planes $C_{R1},C_{R2},C_{R3},C_{R4},C_{R5},C_{R6},C_{R7},C_{R8}$ are obtained.

Step2: extract the original image feature values and decrypt $C_{R5}$

The recipient extracts the eigenvalues of the original image stored in the first line of $C_{R5}$ and obtains the $key_{1}$ according to the Equation 3 (a). The decrypted image $Q_{R5}$ of the fifth layer plane can be obtained. The specific operation is described as

(Equation 17) $$Q_{R5}=Chain\_decrypt\left(C_{R5},key_{1}\right)$$

Step3: chain decryption of the remaining bit planes decomposition

(Equation 18) $$\left\{ \begin{array}{c} Q_{R6}=Chain\_decrypt\left(C_{R6},key_{5}\right)\\ Q_{R7}=Chain\_decrypt\left(C_{R7},key_{6}\right)\\ Q_{R8}=Chain\_decrypt\left(C_{R8},key_{7}\right)\\ Q_{R1}=Chain\_decrypt\left(C_{R1},key_{8}\right)\\ Q_{R2}=Chain\_decrypt\left(C_{R2},key_{8}\right)\\ Q_{R3}=Chain\_decrypt\left(C_{R3},key_{8}\right)\\ Q_{R4}=Chain\_decrypt\left(C_{R4},key_{8}\right) \end{array}\right.$$

Step4: recovery bit planes decomposition

(Equation 19) $$Q_{R}=\sum_{i=1}^{8}2^{i-1}Q_{Ri}=2^{0}Q_{R1}+2^{1}Q_{R}2+2^{2}Q_{R3}+2^{3}Q_{R4}+2^{4}Q_{R5}+2^{5}Q_{R6}+2^{6}Q_{R7}+2^{7}Q_{R8}$$

So far, we get the image $Q_{R}$ of *R* channel after chain decryption. Similarly, the decrypted image $Q_{G},Q_{B}$ of the remaining two channels can be obtained, and the image *Q* is obtained after compounding the three channels.

Step5: decrypt random order substitution

After substituting the extracted hash eigenvalue of the original image into the formula Eq. (3)(b), the $key_{2}$ can be obtained. After substituting the $key_{1}$ and $key_{2}$ into the chaotic system, two chaotic sequences *S* and $S^{\prime }$ can be obtained, respectively. The index matrix *I* is obtained after $S^{\prime }$ is sorted by the sort(·) function. The random order substitution decryption formula is given as follows:

(Equation 20) $$BC_{I_{i,j},j}=\left\{ \begin{array}{ll} \left(Q_{I_{i,j},j}-Q_{I_{i,j-1},j-1}-\left\lfloor 2^{32}\times S_{I_{i,j},j}\right\rfloor \right)\mathrm{mod}\;256 & for\;i=1∼W,j=2∼W\\ \left(Q_{I_{i,j},j}-Q_{I_{i-1,W},W}-\left\lfloor 2^{32}\times S_{I_{i,j},j}\right\rfloor \right)\mathrm{mod}\;256 & for\;i=2∼W,j=1\\ \left(Q_{I_{i,j},j}-Q_{I_{H,W},W}+\left\lfloor 2^{32}\times S_{I_{i,j},j}\right\rfloor \right)\mathrm{mod}\;256 & for\;i=1,\;j=1 \end{array}\right.$$

So far, the decrypted image BC can be obtained, which is the cipher image only encrypted by block permutation.

Step6: decrypt block permutation

Deblock permutation is the inverse operation of block permutation encryption. Firstly, pseudo-random sequences are generated through 2D-LSCM chaotic system using the key, followed by the image being segmented into blocks, and then block the image BC. According to the generated sequence, the decryption operations of color component scrambling, positive-negative transformation, block rotation and block inversion, and block permutation are performed in turn. Finally, the decrypted images are combined to obtain the final decrypted image *P*.

### Experimental results and analysis discussion

#### Experimental environment

We utilized a personal computer (PC) equipped with MATLAB R2023a software as our experimental platform. The system was powered by an AMD Ryzen 9 5950X central processing unit (CPU), featuring a clock frequency of 3.88 GHz. The device had 32 GB of memory and a 4TB hard drive, operating on the Windows 10 operating system. USC-SIPI image database was used in the experimental data selection.

#### Experimental results and analysis

##### Histogram analysis

**Figure 9:**
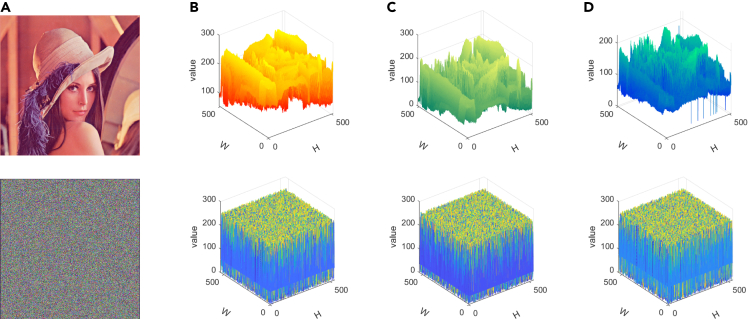
Images before and after encryption and their 3D histograms (A) Plain image and cipher image. (B) Red channel. (C) Green channel. (D) Blue channel.

**Figure 10:**
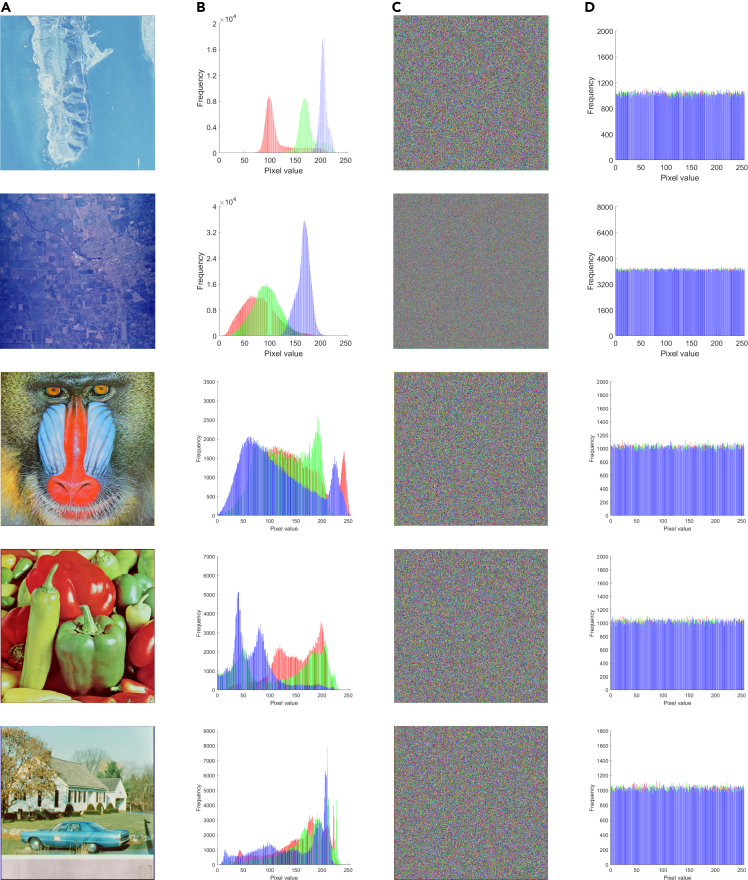
The images before and after encryption and their 2D histogram (A) Plain images. (B) Histogram of (A). (C) Cipher images. (D) Histogram of (C).

Figure 9 displays the 3D visualization of the pixel distribution prior to and following the encryption of three channels, enabling an observation of the cross-plane encryption effect. This visualization serves as a simple yet effective demonstration of the algorithm’s ability to achieve high-performance encryption. Meanwhile, we selected five other images of different types and encrypted them. The renderings and histograms are presented in Figure 10. The original image shows a certain statistical law, while the statistical characteristics of the encrypted image histograms show a noise-like distribution, which well hides the gray value information of the images. This measure strengthens the resilience against statistical analysis attacks.

##### Adjacent pixel correlation analysis

Ordinary images are typically composed of pixels that have a high degree of neighborhood correlation. However, when using a robust encryption algorithm, the encrypted image should ideally have no association between individual pixels and their nearby counterparts. Therefore, a reliable encryption scheme should transform a regular image into an encrypted image with minimal correlation among neighboring pixels.

**Figure 11:**
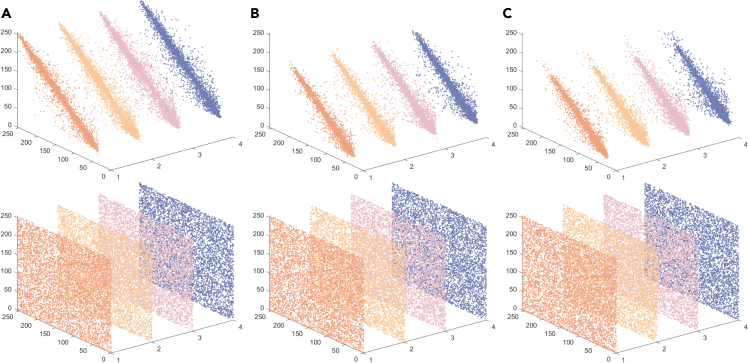
Analysis results of adjacent pixels of plain and cipher images (A) Red channel. (B) Green channel. (C) Blue channel.

To evaluate the correlation between adjacent pixels in both plaintext and cipher images, we took the following measures. First, we randomly selected 3,000 pairs of neighboring pixel points from both the plaintext and cipher images. Secondly, we computed the correlation coefficients of the neighboring pixels in diverse orientations such as horizontal, vertical, diagonal, and anti-diagonal directions individually. In accordance with Equation 21, the correlation coefficients are calculated as follows:

(Equation 21) $$r_{xy}=\frac{\sum_{i=1}^{M}\left(x_{i}-\frac{1}{M}\sum_{j=1}^{M}x_{j}\right)\left(y_{i}-\frac{1}{M}\sum_{j=1}^{M}y_{j}\right)}{\sqrt{\sum_{i=1}^{M}{\left(x_{i}-\frac{1}{M}\sum_{j=1}^{M}x_{j}\right)}^{2}}\sqrt{\sum_{i=1}^{M}{\left(y_{i}-\frac{1}{M}\sum_{j=1}^{M}y_{j}\right)}^{2}}}$$

where $x_{i}$ and $y_{i}$ form the first pair of horizontal/vertical/diagonal/anti-diagonal adjacent pixels and *M* is the total number of horizontal/vertical/diagonal/anti-diagonal adjacent pixels. The correlation between adjacent pixels data of the encrypted image is shown in Figure 11. Figure 11 shows the adjacent pixel distribution of the RGB channels before and after "Lena" image encryption. Experimental data indicate a pronounced contrast in the correlation coefficients between typical images and their encrypted counterparts. In particular, the correlation coefficient for a normal image has a value close to 1, while that for an encrypted image is approximately equal to 0. This highlights the ability of the proposed encryption scheme to generate images with uncorrelated neighboring pixels, emphasizing its resistance to statistical attacks. Therefore, the scheme presented in this study can be considered as highly secure.

##### Differential statistical analysis

Two standards are typically used to measure the dissimilarity between the source image and its encrypted version: the number of pixel change rate (NPCR) and the uniform average change intensity (UACI). In standard disparate attacks, attackers tend to make subtle modifications to the source image and then encrypt the original image using the proposed algorithm. This approach allows them to reveal the underlying relationship between the original and encrypted images. NPCR and UACI criteria are commonly used to evaluate the resistance of an encryption scheme to disparate attacks. The equations for calculating NPCR and UACI are as follows:

(Equation 22) $$\left\{ \begin{array}{l} NPCR=\frac{1}{H\times W}\times \sum_{i=1}^{H}\sum_{i=1}^{W}D\left(i,j\right)\times 100\%\\ ACI=\frac{1}{H\times W}\times \sum_{i=1}^{H}\sum_{j=1}^{W}\frac{\left|v_{1}\left(i,j\right)-v_{2}\left(i,j\right)\right|}{255}\times 100\% \end{array}\right.$$

where H×W represents the size of the image, and $v_{1},v_{2}$ is the cipher image before and after changing one pixel of the plaintext image. *D* can be defined by the Equation 23.

(Equation 23) $$D=\left\{ \begin{array}{ccc} 0, & if & v_{1}\left(i,j\right)=v_{2}\left(i,j\right)\\ 1, & if & v_{0}\left(i,j\right)\neq v_{2}\left(i,j\right) \end{array}\right.$$

**Figure 12:**
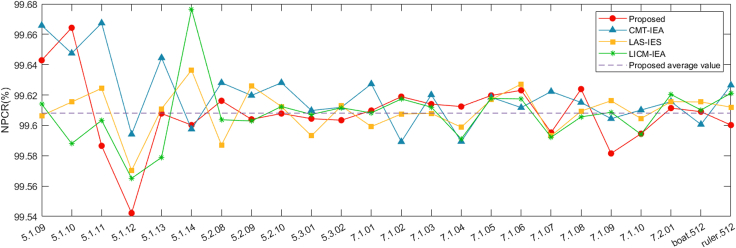
Results of NPCR visualization across different algorithms for comparison

**Figure 13:**
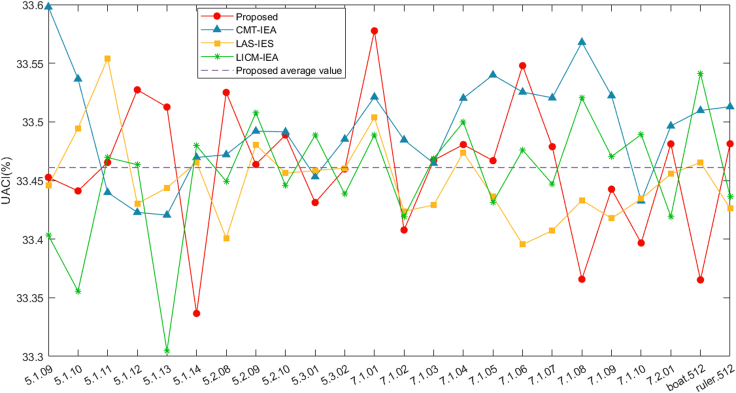
Comparison results of UACI visualization with different algorithms

Tables 2 and 3 show the algorithm’s outcomes as computed per Equation 22. These tables reveal that NPCR and UACI align closely with their expected values of 99.6094% and 33.4635%, respectively. Our algorithm has been further compared with classical algorithms^63^^,^^64^ and other algorithms,^65^ with the comparative results presented in both Figures 12 and 13. The results obtained indicate that the proposed encryption scheme exhibits sensitivity to variations in the source image, allowing the generation of two unique encrypted images even in the presence of a single difference bit. This substantiates the effectuality and robustness of the presented cryptosystem to changes in the input image, increasing its overall reliability and viability for various real-world applications.

**Table 2:** NPCR values of the matching cipher images by distinct algorithms

Images	Description	Size	Type	Proposed	CMT-IEA^63^	LAS-IES^64^	LICM-IEA^65^
5.1.09.tiff	Moon surface	256	gray	99.6429	99.6658	99.6064	99.6140
5.1.10.tiff	Aerial	256	gray	99.6643	99.6475	99.6154	99.5880
5.1.11.tiff	Airplane	256	gray	99.5865	99.6674	99.6244	99.6033
5.1.12.tiff	Clock	256	gray	99.5422	99.5941	99.5703	99.5651
5.1.13.tiff	Resolution chart	256	gray	99.6078	99.6445	99.6109	99.5789
5.1.14.tiff	Chemical plant	256	gray	99.6002	99.5975	99.6364	99.6765
5.2.08.tiff	Couple	512	gray	99.6162	99.6281	99.5870	99.6037
5.2.09.tiff	Aerial	512	gray	99.6040	99.6197	99.6260	99.6029
5.2.10.tiff	Stream and bridge	512	gray	99.6078	99.6281	99.6124	99.6124
5.3.01.tiff	Male	1024	gray	99.6044	99.6098	99.5931	99.6072
5.3.02.tiff	Airport	1024	gray	99.6034	99.6119	99.6128	99.6116
7.1.01.tiff	Truck	512	gray	99.6098	99.6273	99.5992	99.6082
7.1.02.tiff	Airplane	512	gray	99.6189	99.5892	99.6075	99.6174
7.1.03.tiff	Tank	512	gray	99.6140	99.6201	99.6079	99.6120
7.1.04.tiff	Car and APCs	512	gray	99.6124	99.5894	99.5988	99.5911
7.1.05.tiff	Truck and APCs	512	gray	99.6197	99.6185	99.6170	99.6178
7.1.06.tiff	Truck and APCs	512	gray	99.6231	99.6117	99.6272	99.6174
7.1.07.tiff	Tank	512	gray	99.5953	99.6223	99.5931	99.5922
7.1.08.tiff	APC	512	gray	99.6239	99.6151	99.6094	99.6056
7.1.09.tiff	Tank	512	gray	99.5815	99.6044	99.6162	99.6086
7.1.10.tiff	Car and APCs	512	gray	99.5945	99.6101	99.6045	99.5941
7.2.01.tiff	Airplane(U-2)	1024	gray	99.6114	99.6156	99.6156	99.6204
boat.512.tiff	Fishing boat	512	gray	99.6090	99.6006	99.6154	99.6101
ruler.512.tiff	Pixel ruler	512	gray	99.6002	99.6265	99.6120	99.6212

**Table 3:** UACI values of the matching cipher images by distinct algorithms

Images	Description	Size	Type	Proposed	CMT-IEA^63^	LAS-IES^64^	LICM-IEA^65^
5.1.09.tiff	Moon surface	256	gray	33.4527	33.5980	33.4456	33.4032
5.1.10.tiff	Aerial	256	gray	33.4411	33.5366	33.4946	33.3557
5.1.11.tiff	Airplane	256	gray	33.4654	33.4398	33.5541	33.4696
5.1.12.tiff	Clock	256	gray	33.5273	33.4228	33.4302	33.4634
5.1.13.tiff	Resolution chart	256	gray	33.5126	33.4205	33.4438	33.3046
5.1.14.tiff	Chemical plant	256	gray	33.3366	33.4696	33.4655	33.4796
5.2.08.tiff	Couple	512	gray	33.5251	33.4720	33.4008	33.4493
5.2.09.tiff	Aerial	512	gray	33.4637	33.4921	33.4804	33.5077
5.2.10.tiff	Stream and bridge	512	gray	33.4888	33.4914	33.4563	33.4457
5.3.01.tiff	Male	1024	gray	33.4312	33.4532	33.4585	33.4886
5.3.02.tiff	Airport	1024	gray	33.4598	33.4853	33.4605	33.4384
7.1.01.tiff	Truck	512	gray	33.5776	33.5212	33.5037	33.4890
7.1.02.tiff	Airplane	512	gray	33.4078	33.4846	33.4237	33.4190
7.1.03.tiff	Tank	512	gray	33.4673	33.4647	33.4291	33.4689
7.1.04.tiff	Car and APCs	512	gray	33.4806	33.5202	33.4739	33.4997
7.1.05.tiff	Truck and APCs	512	gray	33.4669	33.5400	33.4362	33.4313
7.1.06.tiff	Truck and APCs	512	gray	33.5479	33.5254	33.3954	33.4760
7.1.07.tiff	Tank	512	gray	33.4788	33.5205	33.4073	33.4470
7.1.08.tiff	APC	512	gray	33.3658	33.5678	33.4332	33.5203
7.1.09.tiff	Tank	512	gray	33.4426	33.5223	33.4177	33.4704
7.1.10.tiff	Car and APCs	512	gray	33.3967	33.4325	33.4344	33.4892
7.2.01.tiff	Airplane(U-2)	1024	gray	33.4812	33.4965	33.4556	33.4192
boat.512.tiff	Fishing boat	512	gray	33.3652	33.5097	33.4654	33.5414
ruler.512.tiff	Pixel ruler	512	gray	33.4813	33.5129	33.4262	33.4363

#### Information entropy analysis

The degree of randomness in a system is typically assessed by using the entropy of the information as a standard metric. For an information source *m*, the information entropy H(m) is given by

(Equation 24) $$H\left(m\right)=-\sum_{i=0}^{L}p\left(m_{i}\right)log_{2}p\left(m_{i}\right)$$

where *L* represents the total number of pixels. The probability of $m_{i}$ is denoted by $p\left(m_{i}\right)$.

Suppose the source sends 256 symbols and we can get the theoretical value H(m)=8 using Equation 24. The closer it is to 8, the less likely it is that an attacker will be able to successfully decode the encrypted image. The Lena image is used as the experimental image of information entropy (size: 256 × 256, type: color). Table 4 compares the entropy values and shows that the experimental results are close to 8. This indicates that the proposed algorithm has good entropy properties.

**Table 4:** The results of information entropy analysis for distinct algorithms

Encryption algorithm	Channel			Average value
	Red	Green	Blue	
Proposed	**7.9993**	**7.9994**	**7.9993**	**7.99933**
Ref. Hua et al.^62^	7.9993	7.9993	7.9993	7.99930
Ref. Kumar et al.^66^	7.9912	7.9914	7.9915	7.99137
Ref. Kadir et al.^67^	7.9278	7.9744	7.9705	7.95757
Ref. Wu et al.^68^	7.9895	7.9894	7.9894	7.98943
Ref. Zhou et al.^69^	7.9992	7.9994	7.9993	7.99930

#### Image quality analysis

In the realm of image processing, peak signal to noise ratio (PSNR) and structural similarity (SSIM) are served as standard metrics for assessing the quality of encryption. The mean square error (MSE) is part of the PSNR, defined as

(Equation 25) $$\left\{ \begin{array}{l} MSE=\frac{1}{H\times W}\sum_{H}^{i=1}\sum_{W}^{j=1}{\left(X\left(i,j\right)-Y\left(i,j\right)\right)}^{2}\\ PSNR=10\times \log_{10}\left(\frac{Q^{2}}{MSE}\right) \end{array}\right.$$

where MSE represents the mean square error between the plaintext image *X* and the cipher image *Y*. The vertical extent of the image is represented by *H*, and the horizontal dimension of the image is denoted by *W*. The pixel level of the image is denoted by *Q*. SSIM is a measure of the similarity between two images, explained as

(Equation 26) $$SSIM\left(X,Y\right)=\frac{\left(2\mu_{X}\mu_{Y}+{\left(0.01L\right)}^{2}\right)\left(2\sigma_{XY}+{\left(0.03L\right)}^{2}\right)}{\left(\mu_{X}^{2}+\mu_{Y}^{2}+{\left(0.01L\right)}^{2}\right)\left(\sigma_{X}^{2}+\sigma_{Y}^{2}+{\left(0.03L\right)}^{2}\right)}$$

where the mean values of image *X* is denoted by $\mu_{X}$ and the mean values of image *Y* is denoted by $\mu_{Y}$. The standard deviation of image *X* is denoted by $\sigma_{X}$, the standard deviation of image *Y* is represented by $\sigma_{Y}$, and *L* indicates the dynamic amplitude of pixel values.

As shown in the Table 5, the values of MSE, PSNR, and SSIM can be determined using mathematical Equations 25 and 26. Ideally, the PSNR of an encrypted image should be around 10 dB, while the SSIM should be between −1 and 1. An absolute SSIM value close to 1 indicates excellent similarity between the images being compared. Therefore, after encryption, variations in SSIM should fluctuate around 0.

**Table 5:** PSNR, MSE, and SSIM values

Images	Description	Size	Type	PSNR(dB)	Ref.^70^	MSE	Ref.^70^	SSIM	Ref.^70^
5.1.09.tiff	Moon surface	256	gray	10.1586	10.1588	6269.29	6269.10	0.0103	0.0098
5.1.10.tiff	Aerial	256	gray	9.2692	9.2858	7694.17	7664.70	0.0099	0.0098
5.1.11.tiff	Airplane	256	gray	7.7554	7.7187	10902.98	10995.00	0.0103	0.0098
5.1.12.tiff	Clock	256	gray	7.2710	7.2913	12189.26	12133.00	0.0081	0.0107
5.1.13.tiff	Resolution chart	256	gray	4.9404	4.9343	20846.78	20876.00	0.0092	0.0064
5.1.14.tiff	Chemical plant	256	gray	9.2195	9.2311	7782.77	7762.00	0.0115	0.0091
5.2.08.tiff	Couple	512	gray	9.6047	9.6319	7122.20	7077.70	0.0095	0.0097
5.2.09.tiff	Aerial	512	gray	8.2012	8.2019	9839.26	9837.60	0.0089	0.0091
5.2.10.tiff	Stream and bridge	512	gray	8.7573	8.7508	8656.67	8669.70	0.0100	0.0085
5.3.01.tiff	Male	1024	gray	8.0013	8.0033	10310.87	10298.00	0.0080	0.0086
5.3.02.tiff	Airport	1024	gray	8.7334	8.7449	8704.47	8681.50	0.0091	0.0091
7.1.01.tiff	Truck	512	gray	9.9018	9.9244	6651.21	6616.70	0.0104	0.0106
7.1.02.tiff	Airplane	512	gray	8.9647	8.9790	8253.05	8225.90	0.0104	0.0110
7.1.03.tiff	Tank	512	gray	10.1862	10.1819	6229.64	6237.20	0.0104	0.0108
7.1.04.tiff	Car and APCs	512	gray	9.8068	9.8081	6798.26	6796.20	0.0101	0.0109
7.1.05.tiff	Truck and APCs	512	gray	9.6001	9.6067	7129.70	7118.80	0.0103	0.0106
7.1.06.tiff	Truck and APCs	512	gray	9.1130	9.1258	7975.85	7952.50	0.0103	0.0095
7.1.07.tiff	Tank	512	gray	10.0470	10.0528	6432.49	6424.00	0.0106	0.0103
7.1.08.tiff	APC	512	gray	10.3267	10.3206	6031.23	6039.80	0.0113	0.0109
7.1.09.tiff	Tank	512	gray	9.8454	9.8368	6738.12	6751.60	0.0107	0.0107
7.1.10.tiff	Car and APCs	512	gray	10.1809	10.1702	6237.23	6252.60	0.0104	0.0108
7.2.01.tiff	Airplane(U-2)	1024	gray	6.3261	6.3214	15152.17	15168.00	0.0048	0.0046
boat.512.tiff	Fishing boat	512	gray	9.3058	9.3009	7629.54	7638.20	0.0097	0.0086
gray21.512.tiff	21 level step wedge	512	gray	7.5706	7.5703	11376.67	11378.00	0.0088	0.0090
ruler.512.tiff	Pixel ruler	512	gray	4.7649	4.7657	21706.57	21702.00	0.0087	0.0075

#### Key space

The key space denotes the encompassing set of all conceivable keys that can be utilized for key generation. The magnitude of the key space is contingent upon the length of secure keys and serves as a critical determinant of the strength of a cryptosystem. It is one of the most important parameters in the overall assessment of the robustness and reliability of a cryptographic system. The image encryption algorithm proposed in this paper utilizes a 2D discrete chaotic system, and the expression of its key space can be given as S∈{a,b,θ,MD5}, where a,b,θ are the key parameters with precision $10^{-16}$ and MD5 are the hash value introduced to enhance the key space, which can generate a 128-bit hash. The approximate estimation of the key space size for this encryption scheme is $10^{3\times 16}\times 2^{128}\approx 2^{287}$. By analyzing Table 6, it becomes evident that our proposed encryption scheme not only demonstrates a notable advantage in terms of the key space over existing schemes but also contributes to the enhanced resilience of our encryption algorithm against various types of attacks.

**Table 6:** Table of key space comparisons

This Article	Ref.^71^	Ref.^72^	Ref.^73^	Ref.^74^
287	128	166	154	224

#### Sensitivity analysis

This section analyses the performance sensitivity of the algorithm separately for both key and plaintext. It is essential that security algorithms have a high level of sensitivity. This implies that even minute alterations in the encryption or decryption process, such as modifications to the key or variations in the plaintext information, will result in incorrect outcomes.

##### Key sensitivity analysis

**Figure 14:**
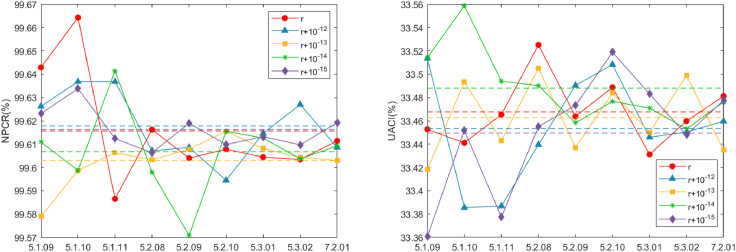
Comparison conclusions of NPCR and UACI presentation with various levels of disturbance

Key sensitivity analysis is performed by comparing the resulting ciphertexts when the identical image is encrypted using two comparable keys. Our study examines the difference in the resulting ciphertext obtained from encrypting with the actual key and encryption using an additional key that contains slight perturbations. The difference between these two results is then evaluated using NPCR and UACI, as calculated via Equation 22, with the consequences presented in Table 7. Interestingly, the consequences express that, even when the key is subjected to slight perturbations, the resulting NPCR and UACI values of the commensurating ciphertext are close to the ideal values of 99.6094% and 33.4635%, respectively. Figure 14 shows the statistical results of NPCR and UACI under different disturbance parameters. In addition, it can be clearly seen from Figure 14 that NPCR and UACI are very close to the ideal values, indicating that the proposed algorithm has good key sensitivity, so it can effectively resist differential attacks and chosen-plaintext attacks.

**Table 7:** Comparison of average encryption times

	Encryption time (s)	Machine specs (CPU and RAM)
Proposed	**2.522390**	$3.88\;\text{GHz}\;{\text{AMD}}^{®},32\;\text{GB}$
Ref. Alexan et al.^75^	2.750966	$3.4\;\text{GHz}\;{\text{Intel}}^{®}\;{\text{Core}}^{\text{TM}}\;i7\;8\;\text{GB}$
Ref. Alexan et al.^76^	2.582389	$2.9\;\text{GHz}\;{\text{Intel}}^{®}\;{\text{Core}}^{\text{TM}}\;i9\;32\;\text{GB}$
Ref. Xu et al.^77^	4.980000	$2.5\;\text{GHz}\;{\text{AMD}}^{®},4\;\text{GB}$

##### Plaintext sensitivity analysis

**Figure 15:**
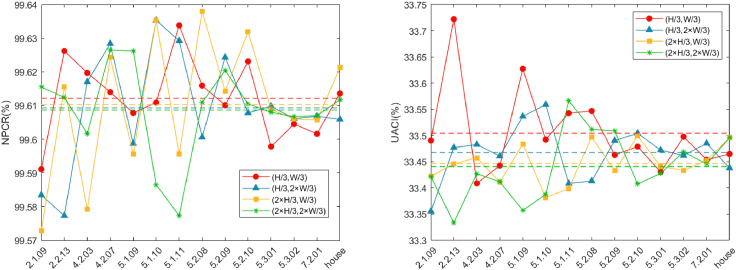
Comparison conclusions of NPCR and UACI presentation with different locations

Plaintext sensitivity refers to the degree of change in the resulting ciphertext when the plaintext pixels are changed. If an algorithm ignores plaintext sensitivity, it becomes vulnerable to attacks that exploit the comparison between plaintext and ciphertext pairs. Consequently, the measure of plaintext sensitivity is a crucial element in determining the robustness of the algorithm against plaintext attacks. In this section, we analyze the sensitivity of the proposed algorithm to ordinary images by adding 1 to the pixel values of ordinary images at (H/3,W/3), (H/3,2×W/3), (2×H/3,W/3), and (2×H/3,2×W/3) to calculate the NPCR and UACI. The results are presented in Table 8, and the comparison images are shown in Figure 15; the dotted line in the figure is the average value of NPCR and UACI for the corresponding color. As indicated in Table 8, when the pixel values at the selected locations are varied by 1, the corresponding encrypted images exhibit a remarkable average NPCR score of 99.6101% compared to the original ciphertext, which is close to the ideal value of 99.6094%. Additionally, an average UACI score of 33.4743% is observed, which is also close to the desirable UACI value of 33.4635%. Based on the results, we can observe that the proposed algorithm makes the cryptographic images susceptible to significant modification, making it insurmountable for attackers to compromise the system by comparing the ciphertexts. Consequently, the proposed algorithm is sufficiently equipped to withstand various types of plaintext attacks.

**Table 8:** Key sensitivity test results

Images	Description	Size	Type	0		$10^{-12}$		$10^{-13}$		$10^{-14}$		$10^{-15}$	
				NPCR	UACI	NPCR	UACI	NPCR	UACI	NPCR	UACI	NPCR	UACI
5.1.09.tiff	Moon surface	256	gray	99.6429	33.4527	99.6262	33.5135	99.5789	33.4181	99.6109	33.5142	99.6231	33.3606
5.1.10.tiff	Aerial	256	gray	99.6643	33.4411	99.6368	33.3855	99.5987	33.4934	99.5987	33.5587	99.6338	33.4519
5.1.11.tiff	Airplane	256	gray	99.5865	33.4654	99.6368	33.3868	99.6063	33.4430	99.6414	33.4939	99.6124	33.3773
5.2.08.tiff	Couple	512	gray	99.6162	33.5251	99.6071	33.4395	99.6033	33.5052	99.5979	33.4903	99.6063	33.4551
5.2.09.tiff	Aerial	512	gray	99.6040	33.4637	99.6086	33.4904	99.6078	33.4370	99.5708	33.4582	99.6189	33.4731
5.2.10.tiff	Stream and bridge	512	gray	99.6078	33.4888	99.5945	33.5082	99.6155	33.4839	99.6155	33.4767	99.6098	33.5189
5.3.01.tiff	Male	1024	gray	99.6044	33.4312	99.6146	33.4459	99.6081	33.4501	99.6126	33.4708	99.6132	33.4830
5.3.02.tiff	Airport	1024	gray	99.6034	33.4598	99.6270	33.4504	99.6043	33.4988	99.6037	33.4530	99.6097	33.4479
7.2.01.tiff	Airplane(U-2)	1024	gray	99.6114	33.4812	99.6086	33.4596	99.6030	33.4348	99.6092	33.4762	99.6191	33.4776

#### The complexity and execution time analysis

We gauge the complexity of the proposed algorithm by measuring the computational time required for encryption and assess its suitability for real-time applications. In this encryption scheme, the most critical four steps are chaotic sequence generation, image scrambling, replacement, and bit-level chain diffusion. Their complexity is O(7×H×W+3/8×H×W), O(7/8×H×W), O(H×W), and O(8×H×W), respectively. Thus, the total complexity is O(8×H×W). Table 9 provides a comparison of encryption times between our proposed algorithm and corresponding algorithms in the literature. It is noteworthy that the encryption time data in Table 9 were obtained using computers with varying processing capabilities and memory configurations to comprehensively evaluate algorithm performance. Such comparisons aid in determining which encryption algorithm is best suited to meet security and performance requirements under specific hardware and environmental conditions.

**Table 9:** Plaintext sensitivity test results

Images	Description	Size	Type	(H/3,W/3)		(H/3,2 × W/3)		(2 × H/3,W/3)		(2 × H/3,2 × W/3)	
				NPCR	UACI	NPCR	UACI	NPCR	UACI	NPCR	UACI
2.1.09.tiff	San Diego(Point Loma)	512	color	99.5911	33.4904	99.5834	33.3552	99.5728	33.4221	99.6155	33.4213
2.2.13.tiff	Stockton	1024	color	99.6262	33.7220	99.5773	33.4769	99.6155	33.4456	99.6124	33.3336
4.2.03.tiff	Mandrill	512	color	99.6197	33.4086	99.6170	33.4828	99.5792	33.4577	99.6017	33.4272
4.2.07.tiff	Peppers	512	color	99.614	33.4424	99.6284	33.4607	99.6243	33.4108	99.6265	33.4115
5.1.09.tiff	Moon surface	256	gray	99.6078	33.6272	99.5987	33.5365	99.5956	33.4835	99.6262	33.3571
5.1.10.tiff	Aerial	256	gray	99.6109	33.4922	99.6353	33.5588	99.6353	33.3817	99.5865	33.3876
5.1.11.tiff	Airplane	256	gray	99.6338	33.5427	99.6292	33.4084	99.5956	33.3985	99.5773	33.5661
5.2.08.tiff	Couple	512	gray	99.6159	33.5467	99.6006	33.4131	99.638	33.4967	99.6109	33.5117
5.2.09.tiff	Aerial	512	gray	99.6101	33.4628	99.6243	33.4903	99.6143	33.4327	99.6204	33.5087
5.2.10.tiff	Stream and bridge	512	gray	99.6231	33.4788	99.6078	33.5042	99.6319	33.4991	99.6105	33.4073
5.3.01.tiff	Male	1024	gray	99.5978	33.4303	99.6098	33.4721	99.6089	33.4410	99.6080	33.4276
5.3.02.tiff	Airport	1024	gray	99.6045	33.4976	99.6061	33.4617	99.6060	33.4332	99.6067	33.4688
7.2.01.tiff	Airplane(U-2)	1024	gray	99.6016	33.4537	99.6068	33.4851	99.6057	33.4522	99.6070	33.4456
house.tiff	House	512	color	99.6136	33.4647	99.6059	33.4378	99.6212	33.4957	99.6117	33.4957

#### Robustness analysis

Robustness is an important index in the evaluation of image encryption algorithm. It measures whether the encryption algorithm can effectively protect the content of the image from damage or leakage in the face of various interference noises. In the real world, images may be affected by a variety of disturbances. Therefore, it is very important to analyze and evaluate the anti-interference ability of image encryption algorithm. In this section, salt and pepper noise and occlusion attack are selected for analysis.

##### Salt and pepper noise analysis

**Figure 16:**
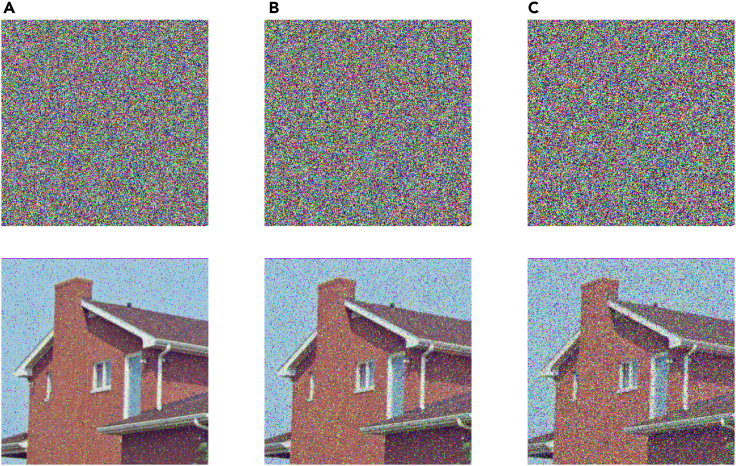
The cipher and decryption image after adding salt and pepper noise (A) Add 5% salt and pepper noise. (B) Add 10% salt and pepper noise. (C) Add 20% salt and pepper noise.

Separately add 5%, 10%, and 20% salt and pepper noise into the plaintext image. We can see from Figure 16 that the image adding noise can still have effective recognizable image information after decryption.

##### Occlusion attack analysis

**Figure 17:**
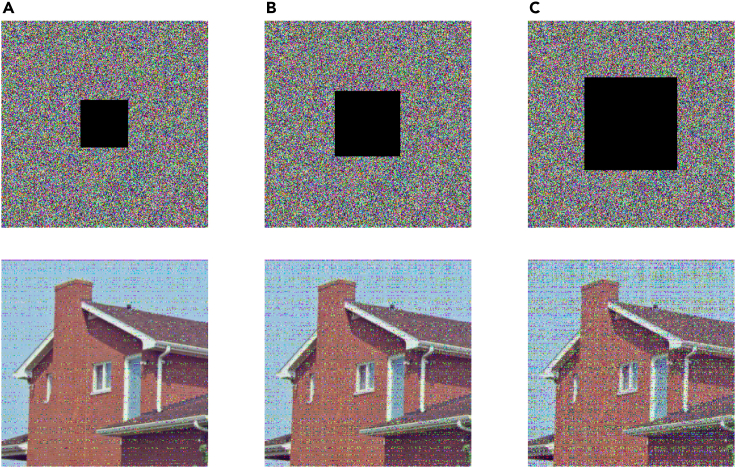
The cipher and decryption image after adding occlusion noise (A) Add 56×56 occlusion noise. (B) Add 81×81 occlusion noise. (C) Add 114×114 occlusion noise.

Respectively add occlusion noise, whose sizes are 56×56, 81×81, and 114×114, into cipher image, and we can see from Figure 17 that the image adding noise can still have effective recognizable image information after decryption.

### Conclusion

This paper presents a comprehensive encryption algorithm for enhancing image security by combining chaos-based block permutation and bit planes chain diffusion. Our approach aims to improve the security of image encryption, enhance its ability to withstand cryptographic attacks in existing computing environments, and ensure secure communication of digital images in networked environments. To begin, we propose a novel block permutation encryption algorithm that employs chaotic sequences to perform various operations on the plaintext image, such as block scrambling, rotation, inversion, positive/negative transformation, and color component transformation. These operations generate an intermediate cipher image, effectively safeguarding the confidentiality of the image. We introduce the random order substitution method, which further increases the difficulty for the attacker to crack the ciphertext. Last but not least, we utilize the hierarchical structure of the bit plane and chain diffusion to generate the final ciphertext. By incorporating these mechanisms, the avalanche effect is enhanced, thus increasing the security of the encryption. Experimental results demonstrate that our proposed algorithm has high security and robustness, making it highly resistant to various cryptographic attacks. Therefore, the image encryption algorithm reported in this paper is a preferred secure communication technology solution, which has a broad application prospect in the secure transmission of multimedia information in big data environments, etc.

### Limitations of the study

There may be some possible limitations in this study. In robustness analysis, there is a very small probability of damaging the original hidden key, resulting in the ciphertext not being able to be restored.

## STAR★Methods

### Key resources table

**Table:** 

REAGENT or RESOURCE	SOURCE	IDENTIFIER
**Software and algorithms**		

Matlab R2023a	MathWorks.Inc	https://ww2.mathworks.cn/products/matlab.html
The USC-SIPI Image Database	University of Southern California	https://sipi.usc.edu/database/database.php

### Resource availability

#### Lead contact

Further information for resources and materials should be directed to and will be fulfilled by the lead contact, Dr. Heping Wen (wenheping@uestc.edu.cn).

#### Materials availability

This study did not generate new unique reagents.

#### Data and code availability

• All experimental data is clearly explained in this paper.
• This paper does not report original code.
• Any additional information required to reanalyze the data reported in this paper is available from the lead contact upon request.

### Method details

In this study, a PC equipped with MATLAB R2023a software was used as the experimental platform. The system is driven by AMD Ryzen 9 5950 X CPU with a clock frequency of 3.88 GHz. The device has 32GB memory and 4TB hard disk and runs on Windows 10 operating system. The experimental data were selected from the USC-SIPI image database. All the software and data involved can be publicly available in the key resources table.

### Quantification and statistical analysis

#### Adjacent pixel correlation analysis

The correlation coefficients are calculated as follows:

$$r_{xy}=\frac{\sum_{i=1}^{M}\left(x_{i}-\frac{1}{M}\sum_{j=1}^{M}x_{j}\right)\left(y_{i}-\frac{1}{M}\sum_{j=1}^{M}y_{j}\right)}{\sqrt{\sum_{i=1}^{M}{\left(x_{i}-\frac{1}{M}\sum_{j=1}^{M}x_{j}\right)}^{2}}\sqrt{\sum_{i=1}^{M}{\left(y_{i}-\frac{1}{M}\sum_{j=1}^{M}y_{j}\right)}^{2}}}$$

where $x_{i}$ and $y_{i}$ form the first pair of horizontal/vertical/diagonal/anti-diagonal adjacent pixels and *M* is the total number of horizontal/vertical/diagonal/anti-diagonal adjacent pixels.

#### Differential statistical analysis

Two standards are typically used to measure the dissimilarity between the source image and its encrypted version: the number of pixel change rate (NPCR) and the uniform average change intensity (UACI). In standard disparate attacks, attackers tend to make subtle modifications to the source image and then encrypt the original image using the proposed algorithm. This approach allows them to reveal the underlying relationship between the original and encrypted images. NPCR and UACI criteria are commonly used to evaluate the resistance of an encryption scheme to disparate attacks. The equations for calculating NPCR and UACI are as follows:

$$\left\{ \begin{array}{c} NPCR=\frac{1}{H\times W}\times \sum_{i=1}^{H}\sum_{i=1}^{W}D\left(i,j\right)\times 100\%\\ UACI=\frac{1}{H\times W}\times \sum_{i=1}^{H}\sum_{j=1}^{W}\frac{\left|v_{1}\left(i,j\right)-v_{2}\left(i,j\right)\right|}{255}\times 100\% \end{array}\right.$$

where H×W represents the size of the image, $v_{1},v_{2}$ is the cipher image before and after changing one pixel of the plaintext image. *D* can be defined:

$$D=\left\{ \begin{array}{cc} 0, & if\;v_{1}\left(i,j\right)=v_{2}\left(i,j\right)\\ 1, & if\;v_{0}\left(i,j\right)\neq v_{2}\left(i,j\right) \end{array}\right.$$

#### Information entropy analysis

The degree of randomness in a system is typically assessed by using the entropy of the information as a standard metric. For an information source *m*, the information entropy H(m) is given by:

$$H\left(m\right)=-\sum_{i=0}^{L}p\left(m_{i}\right)log_{2}p\left(m_{i}\right)$$

where *L* represents the total number of pixels. The probability of $m_{i}$ is denoted by $p\left(m_{i}\right)$.

#### Image quality analysis

In the realm of image processing, Peak Signal to Noise Ratio (PSNR) and Structural Similarity (SSIM) are served as standard metrics for assessing the quality of encryption. The Mean Square Error (MSE) is part of the PSNR, defined as:

$$\left\{ \begin{array}{l} MSE=\frac{1}{H\times W}\sum_{H}^{i=1}\sum_{W}^{j=1}{\left(X\left(i,j\right)-Y\left(i,j\right)\right)}^{2}\\ PSNR=10\times \log_{10}\left(\frac{Q^{2}}{MSE}\right) \end{array}\right.$$

where MSE represents the mean square error between the plaintext image *X* and the ciphertext image *Y*. The vertical extent of the image is represented by *H*, the horizontal dimension of the image is denoted by *W*. The pixel level of the image is denoted by *Q*. SSIM is a measure of the similarity between two images, explained as:

$$SSIM\left(X,Y\right)=\frac{\left(2\mu_{X}\mu_{Y}+{\left(0.01L\right)}^{2}\right)\left(2\sigma_{XY}+{\left(0.03L\right)}^{2}\right)}{\left(\mu_{X}^{2}+\mu_{Y}^{2}+{\left(0.01L\right)}^{2}\right)\left(\sigma_{X}^{2}+\sigma_{Y}^{2}+{\left(0.03L\right)}^{2}\right)}$$

where the mean values of image *X* is denoted by $\mu_{X}$, the mean values of image *Y* is denoted by $\mu_{Y}$. The standard deviation of image *X* is denoted by $\sigma_{X}$, the standard deviation of image *Y* is represented by $\sigma_{Y}$ and *L* indicates the dynamic amplitude of pixel values.
